# Editorial: Advances in understanding the pain chronification mechanisms

**DOI:** 10.3389/fpain.2023.1228264

**Published:** 2023-06-09

**Authors:** Marco Pagliusi, Ivan Bonet, Cesar Sartori

**Affiliations:** ^1^ Department of Pharmacology, University of São Paulo, Ribeirão Preto, Brazil; ^2^ Department of Oral & Maxillofacial Surgery, University of California, San Francisco, CA, United States; ^3^ Department of Functional and Structural Biology, State University of Campinas, Campinas, Brazil

**Keywords:** pain chronification, chronic pain, co-morbidities, pain management, pain mechanism

**Editorial on the Research Topic** Advances in understanding the pain chronification mechanisms

Chronic pain (CP) is a highly prevalent social issue that negatively impacts the quality of life of affected individuals, imposing a great economic burden on the medical system. It is well known that central and peripheral mechanisms are involved in the pain chronification process, although our understanding of the mechanisms, risk factors, and sociodemographic characteristics underlying the development of chronic pain remains limited. These insights are essential to managing this condition in the clinical or public policy context and for the development of new preventive and therapeutic approaches, both pharmacological and non-pharmacological. In this research topic, we could assess and discuss part of these aspects, helping to shed light on the theme.

Eller et al. aimed to investigate possible epigenetic hallmarks that could differentiate patients showing acute low back pain (aLBP) vs. chronic low back pain (cLBP). In this study, the researchers followed the patients for 24 weeks and differentiated those who developed chronic pain and those who did not based on this follow-up. Interestingly, they observed that patients who developed cLBP showed increased pain burden across multiple subscales when compared to aLBP patients. They also observed that cLBP patients showed decreased global DNA methylation when compared to aLBP patients and healthy controls, which is correlated with a higher expression of IL-2 mRNA in the cLBP group when compared to others.

The study carries out by Henri et al. aimed to investigate the phenomenon of pleasant pain relief (PPR), where the withdrawal of a painful stimulus elicits a pleasant sensation. The PPR can also be triggered during the cold pressor test (CPT), which has been widely used to study diffuse noxious inhibitory control, where the pain is inhibited by another painful stimulus. Considering that chronic pain patients have an impaired inhibitory control of pain, assessing the variability of the individual response during this procedure is very relevant. In this study, the authors brought to light that both pain experience and pain sensitization may underlie individual differences in PPR responses.

This research topic also features the hypothesis-based article authored by Macionis which aimed to discuss that the transition from acute to chronic pain may involve the development or aggravation of compressive proximal neural lesions. The author presents a complete manuscript from the definition of proximal neural lesions to the implications for chronic pain management, hypothesizing that there is a common neuropathic etiology of all types of general chronic pain.

Finally, Hanson et al. aimed to examine the sociodemographic characteristics of Veterans in the Phoenix VA Health Care System who have back pain. They showed that Hispanic/Latinx, Black/African American, or Native American/Alaskan were under-referred to pain clinics and, on the other hand, those patients with depressive and opioid use disorders, were more likely to be referred to the pain clinic.

The editors hope you enjoy this special issue and that it will be useful and insightful to your clinical practice and research.

